# Chromosome-level genome assemblies of Thai cassava ecotypes (*Manihot esculenta* & *Manihot glaziovii*)

**DOI:** 10.1038/s41597-025-05998-3

**Published:** 2025-09-30

**Authors:** Sebastian Beier, Marie Elizabeth Bolger, Yindee Chanvivattana, Anthony Michael Bolger, Maximilian Heinrich-Wilhelm Schmidt, Oranuch Leelapon, Rungroj Rakmit, Kulnida Changmai, Ratiporn Ruayreun, Sukanya Srithundon, Duangjit Totaiya, Boriphat Sitchanukrit, Björn Usadel, Prapit Wongtiem, Suwaluk Amawan, Tobias Wojciechowski

**Affiliations:** 1 Institute of Bio- and Geosciences (IBG-4 Bioinformatics), CEPLAS, BIOSC, Forschungszentrum Jülich GmbH, Wilhelm Johnen Straße, Jülich, Germany; 2 National Biobank of Thailand (NBT), National Center for Genetic Engineering and Biotechnology (BIOTEC), National Science and Technology Development Agency (NSTDA), Pathum Thani, Thailand; 3 Department of Grapevine Breeding, Hochschule Geisenheim University, Geisenheim, Germany; 4 Heinrich-Heine University Düsseldorf, Faculty of Mathematics and Natural Sciences, Institute for Biological Data Science, CEPLAS, Düsseldorf, Germany; 5 Rayong Field Crops Research Center, Department of Agriculture, 320 Huaypong, Muang, Rayong, 21150 Thailand; 6 Institute of Bio- and Geosciences (IBG-2 Plant Sciences), Jülich Plant Phenotyping Center, Forschungszentrum Jülich GmbH, Wilhelm Johnen Straße, Jülich, Germany

**Keywords:** Plant genetics, Agriculture

## Abstract

Cassava is a vital staple crop, yet genomic resources for diverse ecotypes, particularly from key regions, remain limited. To address this, we generated high-quality genome assemblies for nine Thai *M. esculenta* cultivars and one wild relative, *Manihot glaziovii*. The sequencing strategy combined Oxford Nanopore long reads for initial assembly with Illumina short reads for polishing and quality assessment. For five of the genotypes, extensive RNA-Seq data from various tissues and developmental stages were also produced to guide gene annotation. We provide detailed technical validation of the ten genome assemblies, reporting on key metrics of contiguity (N50s from 28.9 to 35.2 Mb), completeness (Complete BUSCO scores from 95.69% to 99.21%), and base-level accuracy (*k*-mer QV scores from 33.47 to 37.67). The final annotated assemblies and all raw sequencing data have been deposited in public archives and are readily accessible. These datasets represent a significant expansion of the genomic toolkit for Asian cassava, providing a foundational resource for future genetic discovery, comparative genomics, and advanced breeding applications.

## Background & Summary

Cassava (*Manihot esculenta* Crantz) is a crop of great importance in global food security, economic development, and industrial applications. This starchy root vegetable serves as a staple food for over 800 million people worldwide, particularly in tropical and subtropical regions^1^. Production reached 315 million tonnes in 2021, marking a 9% increase from 2017 worldwide^2^. Nigeria, the leading producer, accounted for approximately 63 million tonnes, representing 31% of African production and 20% of global production. Notably, the top three producers - Nigeria, the Democratic Republic of Congo, and Thailand - contribute a combined 44% share of global cassava production^2^. The resilience of cassava to challenging growing conditions, including drought and marginal soil, makes it an ideal crop for regions prone to climate variability^3^. This adaptability ensures a stable food supply, contributing significantly to food security in developing countries^4^. Additionally, its industrial applications are diverse, ranging from starch production to biofuels, sweeteners, glues and animal feed, with the global cassava starch market expected to reach USD 99.91 billion by 2032. Cassava’s role in local economies is equally significant, supporting rural development and poverty alleviation by providing income opportunities for smallholder farmers, processors, and traders.

Originating from the southern Amazon basin, where it was likely domesticated thousands of years ago, cassava’s cultivation spread throughout pre-Columbian South America^5,6^. Following European contact, Portuguese traders introduced cassava to Africa, likely starting in the Congo Basin region around the 16th century. Its remarkable adaptability to diverse climates and soils fueled its widespread adoption across the African continent, where it became a fundamental staple crop. Introduction into Asia occurred later, possibly around the 18th century via trade routes, establishing cassava as a vital agricultural commodity in countries like Thailand, Indonesia, and Vietnam. This historical global spread has shaped distinct regional germplasm pools and adaptation strategies.

To fully leverage cassava’s potential and accelerate breeding efforts, comprehensive genomic resources are indispensable. Initial efforts resulted in a draft genome sequence from the Latin American-derived cultivar AM560-2 (originating from Colombia/CIAT breeding programs)^7^, providing a foundational resource for the research community. Subsequent advancements, incorporating long-read sequencing technologies (like PacBio and ONT) and scaffolding methods such as chromatin conformation capture (Hi-C), have significantly enhanced the quality and contiguity of reference genomes^8^. This includes improved chromosome-level assemblies for AM560-2 (e.g., versions v6, v7, v8)^9^ and the African landrace TMEB117 (widely used in IITA breeding programs)^10^.

*Manihot esculenta* is a diploid species (2n = 36) with an estimated haploid genome size of approximately 750 Mbp^11^. A key complicating factor is the genome’s high degree of heterozygosity, often reported to be in the range of 1.0-1.5%^12^. This high level of sequence divergence between homologous chromosomes, a consequence of cassava’s typically outcrossing reproductive system and widespread clonal propagation, poses significant hurdles for genome assembly algorithms. Standard approaches often struggle to differentiate allelic sequences, leading to the artificial merging (collapse) of haplotypes into a single chimeric sequence or the fragmentation of the assembly where divergent alleles are represented as separate contigs. Accurately resolving these haplotypes is critical for understanding allele-specific expression, identifying causal variants for traits, and developing precise breeding strategies.

While reference genomes exist for American and African cassava germplasm, high-quality genomic resources for Asian ecotypes have been lacking. A draft assembly for the Thai cultivar ‘Kasetsart 50’ (KU50)^13^ was an important first step, but a broader, high-quality representation of the diversity within Thailand was needed to empower regional breeding and research efforts. This study addresses this gap by generating and validating ten chromosome-level genome assemblies from a panel of diverse Thai *Manihot esculenta* cultivars and a wild *M. glaziovii* relative. Here, we describe the methods used for plant selection, sequencing, genome assembly, and annotation. We then present a detailed technical validation of the assemblies and gene models, confirming their quality and completeness. The resulting datasets provide a foundational genomic resource for the cassava research community, enabling future studies into the genetic diversity and improvement of this crop.

## Methods

### Plant material

Ten distinct *Manihot* genotypes were selected for genome sequencing to capture genetic diversity related to various traits within cultivated cassava (*Manihot esculenta*) and its wild relative *M. glaziovii*. The panel included the established low-yield, sweet cultivar ‘Hanatee’. Four commercially important Thai cultivars were also sequenced: ‘Kasetsart50’, a widely recommended variety; ‘Rayong9’, noted for high ethanol yield potential; ‘Rayong72’, characterized by high yield, high dry matter, and adaptation to Northeast Thailand; and ‘Rayong90’, known for high root dry matter content.

Additionally, five accessions were collected during a field visit in Northern Thailand (Nan province). These included ‘HighlandRough’ and ‘HighlandSmooth’, which are putative ‘Hanatee’ variants selected specifically for their contrasting rough and smooth bark phenotypes. Two landraces distinguished by their storage root flesh color, ‘WhiteRoot’ and ‘YellowRoot’, were also collected in the tropical rainforests of Narathiwat province in the south of Thailand. Finally, an accession of the wild relative *Manihot glaziovii* (designated ‘*M. glaziovii* WildType’), historically significant in breeding programs (e.g., for rubber traits), was provided by the Rayong Field Crop Research Center. This diverse germplasm set provides a foundation for comparative genomics studies within the *Manihot* genus and especially the cassava ecotypes of Thailand.

### Sample preparation and sequencing

Young leaves from mature cassava plants were collected and flash-frozen. High molecular weight genomic DNA (HMW gDNA) used for Illumina and Oxford Nanopore Technologies (ONT) sequencing was extracted from the leaf tissues using a protocol provided by ONT (https://nanoporetech.com/document/extraction-method/fever-tree-gdna). The concentration and quality of the extracted DNA were assessed using a NanoDrop spectrophotometer and Qubit. Short strands of DNA were removed from the samples using circulomic SRE XL.

#### ONT reads

The HMW gDNA was used for ONT DNA library prep using the SQK-LSK109 kit and sequenced either on a MinION using the FLO-MIN106 flow cell (21 libraries), or on a PromethION using the FLO-PR002 flow cell (19 libraries). Reads were basecalled using Dorado (v0.5.2) with the model r941_prom_sup_g507 which generated 793.4 Gbp in total^14–23^.

#### Illumina short reads

Illumina short-read library was constructed from the HMW gDNA and sequenced on Illumina NextSeq 2000 to generate 150 bp paired-end reads. The short-read sequencing generated approximately 138 Gbp of raw data, consisting of 460.1 million paired-end (2 × 150 bp) reads^14–23^.

#### RNA-seq reads

RNA used for gene prediction was obtained from a time-course experiment on cassava (*Manihot esculenta* and *Manihot glaziovii*) tubers. Total RNA was extracted from tubers at multiple tuber developmental stages and time points. RNA sequencing was performed by an external service provider using Illumina technology. The *Manihot esculenta* cultivar Hanatee was sequenced using 75 bp single-end reads, whereas all other samples (*Manihot esculenta* Kasetsart50, Rayong9, Rayong72, and *Manihot glaziovii* WildType) were sequenced using 150 bp paired-end reads (2 × 150 bp). In total approximately 1898 Gbp of raw data was generated^24–111^.

### Genome size and heterozygosity estimation

The genome characteristics of the ten *Manihot* species, including genome size and heterozygosity were estimated using Illumina short read data and a *k*-mer based approach. A 21-mer frequency distribution was generated with Jellyfish (v2.3.1)^112^, and the genome’s key features were inferred using GenomeScope2 (v2.0)^113^. The haploid genome size of the nine *Manihot esculenta* genotypes was estimated between 556 Mbp and 676 Mbp, with a heterozygosity rate estimated between 1.30% and 1.79%, while the genome size of *Manihot glaziovii* was estimated at 659 Mbp, with a heterozygosity rate at 4.62%.

### *De novo* genome assembly, Ragtag scaffolding and quality assessment

The assembly of cassava genomes was performed using a combination of long-read sequencing data and multiple assembly refinement steps. Oxford Nanopore Technologies (ONT) reads were assembled using Flye (v.2.9.3)^114^ with parameters --read-error 0.03, -m 10000, and NextDenovo (v.2.5.0)^115^ to generate two independent draft assemblies. The completeness and quality of these assemblies were then assessed using Merqury (v.1.3)^116^. To improve the base accuracy, the assemblies were then polished using Medaka (https://github.com/nanoporetech/medaka, v.1.12.0) with ONT read data. After that, Purge_Dups (v.1.2.6)^117^ was applied to both assemblies to reduce redundancy caused by haplotigs. The two purged assemblies for each genome were then merged using QuickMerge (v.0.3)^118^ to generate a consensus genome. To further refine the assembly structure, RagTag (v.2.1.0)^119^ was employed, with the correct submodule first applied using the published South American reference genome of *Manihot esculenta* AM560-2 (GCA_001659605.2), followed by the scaffold submodule to enhance contiguity.

Finally, the NextPolish programme (v.1.4.1)^120^ was used for two rounds of polishing with ONT read data to fill gaps and improve sequence accuracy. Following this, a contaminant screening step was performed. All unplaced contigs were subjected to a blastn (v2.16)^121^ search against the ‘core_nt’ database using the parameters: -max_target_seqs. 1 -evalue 1e-10 -culling_limit 5 -outfmt “6 qseqid sseqid pident length mismatch gapopen qstart qend sstart send evalue bitscore staxids sscinames”. Any contigs whose best hit was not to a plant species were removed. The resulting assemblies provided high-quality genome sequences for all 10 cassava accessions (Table 1)^122–131^.

**Table 1:** Summary of the ten *Manihot* genome assemblies.

Species	Hanatee	HighlandSmooth	HighlandRough	Rayong9	Rayong72	Rayong90	Kasetsart50	WildType	WhiteRoot	YellowRoot
	*Manihot Esculenta*	*Manihot Esculenta*	*Manihot Esculenta*	*Manihot Esculenta*	*Manihot Esculenta*	*Manihot Esculenta*	*Manihot Esculenta*	*Manihot Glazovii*	*Manihot Esculenta*	*Manihot Esculenta*
Genome size(Mb)	545.8	553.4	559.8	552.1	594.5	668.5	587.1	1299.6	570.5	593.5
Chromosome size (Mb)	528.1	529.3	536.8	525.5	567.4	615.3	566.3	727.6	550.3	561.1
GC content (%)	36.98	37.04	37.02	36.97	37.32	37.51	37.27	38.04	37.19	37.21
N50 (Mb)	28.9	29.1	31.1	29.2	31.9	35.2	32.4	33.8	30.0	31.0
N90 (Mb)	26.5	24.6	21.6	23.3	24.7	21.4	25.8	0.098	26.3	25.8
Number of contigs	532	502	559	486	640	435	496	5099	392	638
Complete BUSCO (%)	96.94	96.79	96.04	96.12	97.07	97.04	97.96	99.21	95.69	97.22
Size of repeat sequences (Mb)	303.3	309.0	313.4	300.5	338.8	357.3	328.9	445.6	323.4	328.8
Total gene number	29,284	30,481	30,254	29,876	29,654	32,817	29,953	50,203	30,044	31,276
*K*-mer completeness (%)	72.8236	71.9676	73.1022	72.7629	73.9351	77.2268	75.1543	89.758	74.1047	73.8572
*K*-mer QV	35.4822	35.7945	36.0497	35.8055	35.7121	33.4691	33.9882	37.6718	35.4231	35.3134
Estimated heterozygosity (%)	1.5	1.37	1.31	1.25	1.51	1.34	1.41	4.56	1.39	1.47

### Genome annotation

Repeat elements within each of the ten cassava genome assemblies were identified and masked using the Extensive *de novo* TE Annotator (EDTA, v2.2.1)^132^. The identified repetitive sequences constitute a significant portion of each genome, ranging from 300.5 to 445.6 Mbp per assembly, which represents over 50% of the total genome size (Table 2). In addition to transposable element annotation by EDTA, SSRs were identified using MISA (v2.1)^133^. The continuity of the repeat regions was estimated by calculating the adjusted LAI^134^. Furthermore, to identify potential telomeric repeat sequences and assess chromosome completeness, the ten *Manihot* genome assemblies were analyzed using the tidk (telomere identification toolkit, v0.2.41)^135^ with the motif ‘CCCTAAA’ and a window size of 10 kbp.

**Table 2:** Summary of repeat annotations for ten *Manihot* assemblies.

	Hanatee	HighlandSmooth	HighlandRough	Rayong9	Rayong72	Rayong90	Kasetsart50	WildType	WhiteRoot	YellowRoot
Class 1 elements (Retrotransposons)										
Order LTR retrotransposons										
Gypsy	112.5 Mb (21.3%)	124 Mb (23.42%)	127 Mb (23.66%)	126.2 Mb (24.03%)	152. 5 Mb (26.88%)	94.3 Mb (15.33%)	139.3 Mb (24.59%)	235.4 Mb (32.35%)	137.7 Mb (25.03%)	143.4 Mb (25.56%)
Copia	17.4 Mb (3.29%)	20.4 Mb (3.85%)	18.7 Mb (3.49%)	18.5 Mb (3.53%)	20.4 Mb (3.59%)	19.2 Mb (3.11%)	18.5 Mb (3.27%)	27.6 Mb (3.79%)	18.4 Mb (3.34%)	21 Mb (3.74%)
Unknown	127.4 Mb (24.12%)	116.1 Mb (21.93%)	120 Mb (22.37%)	114.2 Mb (21.72%)	111.8 Mb (19.7%)	113.6 Mb (18.46%)	126.3 Mb (22.3%)	126.7 Mb (17.41%)	122.7 Mb (22.3%)	116.4 Mb (20.74%)
Order LINE	4.2 Mb (0.8%)	5 Mb (0.95%)	4.8 Mb (0.9%)	4.8 Mb (0.93%)	13.8 Mb (2.45%)	89.3 Mb (14.52%)	4.1 Mb (0.73%)	6.2 Mb (0.85%)	6.3 Mb (1.15%)	7.8 Mb (1.38%)
Order SINE	n/a	29 kb (0.01%)	35 kb (0.01%)	n/a	47 kb (0.01%)	n/a	2 kb (0.00%)	45 kb (0.01%)	50 kb (0.01%)	20 kb (0.00%)
Class 2 elements (DNA Transposons)										
Order TIR										
CACTA	2.9 Mb (0.55%)	2.7 Mb (0.51%)	2.4 Mb (0.45%)	2.3 Mb (0.43%)	2.7 Mb (0.47%)	4 Mb (0.65%)	3 Mb (0.52%)	6.3 Mb (0.87%)	2.7 Mb (0.48%)	4.8 Mb (0.86%)
Mutator	8.4 Mb (1.58%)	11.5 Mb (2.18%)	7.9 Mb (1.48%)	5 Mb (0.94%)	5.3 Mb (0.93%)	6.8 Mb (1.11%)	6.8 Mb (1.21%)	9.6 Mb (1.31%)	6.4 Mb (1.15%)	5.5 Mb (0.99%)
hAT	4.9 Mb (0.93%)	2.7 Mb (0.51%)	3.7 Mb (0.68%)	3.4 Mb (0.65%)	5.7 Mb (1%)	3.5 Mb (0.57%)	6.7 Mb (1.19%)	6.9 Mb (0.95%)	2.9 Mb (0.53%)	3.2 Mb (0.58%)
Tc1/Mariner	61 kb (0.01%)	165 kb (0.03%)	59 kb (0.01%)	229 kb (0.04%)	211 kb (0.04%)	81 kb (0.01%)	72 kb (0.01%)	595 kb (0.08%)	874 kb (0.16%)	45 kb (0.01%)
PIF/Harbinger	3.4 Mb (0.64%)	3.8 Mb (0.71%)	4.5 Mb (0.84%)	1.5 Mb (0.28%)	1.2 Mb (0.22%)	2 Mb (0.32%)	1.6 Mb (0.28%)	2.4 Mb (0.33%)	4.9 Mb (0.9%)	2.1 Mb (0.38%)
Order Helitron	973 kb (0.18%)	1 Mb (0.2%)	3.1 Mb (0.58%)	1.5 Mb (0.28%)	1.8 Mb (0.31%)	2.9 Mb (0.47%)	1.5 Mb (0.27%)	2 Mb (0.28%)	1.1 Mb (0.2%)	4.5 Mb (0.8%)
Unclassified elements	19.8 Mb (3.75%)	20.6 Mb (3.89%)	19.6 Mb (3.64%)	22 Mb (4.19%)	21.9 Mb (3.85%)	21.1 Mb (3.42%)	19.3 Mb (3.42%)	21.4 Mb (2.94%)	18.4 Mb (3.35%)	19.2 Mb (3.43%)
Tandem repeats	672 kb	633 kb	654 kb	567 kb	662 kb	624 kb	630 kb	611 kb	616 kb	627 kb
LAI (adjusted)	18.78	19.45	19.17	19.31	19.64	30.61	19.49	19.59	19.37	19.70

The strategy for predicting protein-coding genes varied depending on the availability of transcriptomic data for each genotype. For five genotypes (*Manihot Esculenta* Hanatee, Kasetsart50, Rayong9, Rayong72, and *Manihot Glaziovii* WildType), available RNA-Seq data were utilized. Briefly, individual RNA-Seq library reads were aligned to their respective genome assemblies using HISAT2 (v2.2.1)^136^ with ‘--dta’ parameter. Using Samtools (v1.20)^137^ libraries were combined and coordinate-sorted. Transcripts were then assembled using StringTie2 (v2.2.3)^138^. Separately, deep learning gene predictions were generated using Helixer (v0.3.3)^139^ using the ‘Land plant’ lineage model via its web interface (https://www.plabipd.de/helixer_main.html). The transcript evidence from StringTie2 and the *ab initio* predictions from Helixer were then integrated using Mikado (v2.2.3)^140^ to produce a consolidated, non-redundant set of gene models for these five genotypes. For the remaining five cassava genotypes, where corresponding RNA-Seq data were not generated in this study, protein-coding genes were predicted solely using the deep-learning-based approach implemented in Helixer (v0.3.3), accessed via its web interface.

Functional annotations for the predicted proteomes derived from all ten genotypes were obtained using Mercator4 (v7)^141^ through the Plabipd web platform (https://www.plabipd.de/mercator_main.html). This process incorporated information from ProtScriber (v0.1.6, https://github.com/usadellab/prot-scriber) and Swiss-Prot^142^ to assign functional categories.

The completeness of the predicted protein-coding gene sets for each of the ten genotypes, in terms of expected gene content, was assessed using BUSCO (v5.8.3)^143^ against the eudicotyledons_odb12 lineage dataset. Furthermore, the quality and consistency were evaluated using Mercator4 (v7) and OMArk (v0.3.0, OMAmer v2.0.5)^144,145^ and PSAURON (v1.0.6)^146^. Results of the quality assessments are summarized in Table 3.

**Table 3:** Summary of gene annotations for ten *Manihot* assemblies.

	Hanatee	HighlandSmooth	HighlandRough	Rayong9	Rayong72	Rayong90	Kasetsart50	WildType	WhiteRoot	YellowRoot
Protein-coding genes										
Total gene number	29,284	30,481	30,254	29,876	29,654	32,817	29,953	50,203	30,044	31,276
Total mRNA number	30,286	30,481	30,254	30,634	30,902	32,817	30,796	51,770	30,044	31,276
Mean gene length (bp)	4598	4511	4491	4590	4632	4511	4602	4648	4489	4491
Mean CDS length (bp)	1327	1294	1297	1331	1329	1295	1311	1321	1289	1294
Mean exon number	6.2	6.1	6.1	6.2	6.2	6.1	5.7	6.1	6.1	6.1
Mean intron number	5.2	5.1	5.1	5.2	5.2	5.1	5.1	5.1	5.1	5.1
Complete protein BUSCOs (%)	96.6	94.3	93.6	94.5	95.0	94.6	95.2	97.5	93.0	94.7
Single Omark HOGs (%)	55.96	54.06	55.2	54.39	55.54	51.53	54.65	27.93	54.82	54.55
Duplicated Omark HOGs (%)	39.85	41.86	41.14	41.67	40.39	45.11	42.41	70.38	40.71	42.09
Missing Omark HOGs (%)	4.18	4.09	3.65	3.94	4.07	3.37	2.95	1.69	4.47	3.37
Consistent Omark HOGs (%)	96.28	96.34	96.43	96.4	95.94	96.23	96.33	96.21	96.39	96.1
Inconsistent Omark HOGs (%)	0.76	0.76	0.77	0.84	0.75	0.78	0.76	0.78	0.78	0.89
Likely Contamination Omark HOGs (%)	0	0	0	0	0	0	0	0	0	0
Unknown Omark HOGs (%)	2.96	2.91	2.8	2.76	3.31	2.99	2.91	3.01	2.83	3.01
Mercator4 proteins annotated (%)	96.37	96.31	96.52	96.04	96.22	96.33	95.87	96.02	96.44	96.09
Mercator4 proteins classified (%)	63.57	62.33	62.61	62.91	62.98	62.18	62.66	63.02	62.5	62.19
Mercator4 BINs occupied (%)	94.73	94.46	94.95	94.8	94.7	94.74	94.85	96.43	93.79	95.38
PSAURON score	97.2	97.1	97.2	97.2	97.0	97.3	97.1	97.1	97.1	97.0

## Data Records

The raw sequencing data, including genomic DNA Illumina and ONT reads and RNA-Seq reads, have been deposited at the EMBL-EBI European Nucleotide Archive (ENA) under BioProject number PRJEB89494 (ERP172520)^147^. The genome assemblies of the ten genotypes have been submitted to ENA under the accessions GCA_965363265^122^, GCA_965363285^124^, GCA_965363275^123^, GCA_965363475^125^, GCA_965365905^131^, GCA_965364345^128^, GCA_965364185^126^, GCA_965364695^130^, GCA_965364235^127^, GCA_965364665^129^. The assembled genome, including annotations, is accessible via an interactive Jbrowse2^148^ instance at https://www.plabipd.de/ceplas/?config=cassavastore.json.

## Technical Validation

### Assembly and annotation quality assessment

We assessed the quality and completeness of the ten *Manihot* genome assemblies using DNA sequencing read mapping and Merqury *k*-mer based evaluation. Illumina paired-end reads were mapped using bwa-mem2 (v2.2.1)^149^, while ONT reads were aligned using minimap2 (v2.28)^150^. Across the ten assemblies, mapping rates ranged from 96.11% to 97.11% for Illumina reads and from 92.82% to 98.12% for ONT reads, indicating successful alignment of the majority of sequencing data to each assembly.

**Fig. 1:**
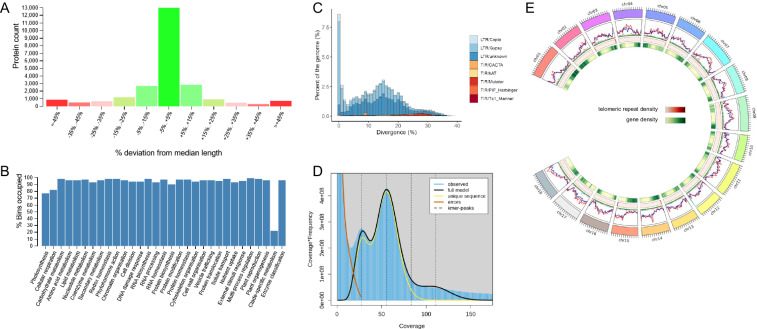
Genome characteristics of *Manihot esculenta* Hanatee. (**A**) Histogram displaying the distribution of proteins grouped by their percentage deviation from the median protein length. (**B**) Histogram showing the percentage of Mercator4 functional BINs occupied by the Hanatee proteins. (**C**) Histogram displaying the divergence of repeat elements by classes and their overall percentage of the genome contribution. (**D**) *k*-mer plot. (**E**) Circos diagram displaying the distribution of different repeat element classes over the individual chromosomes, compared directly to the found telomeric repeats and gene density.

Assembly quality was further evaluated using CRAQ (v1.0.9)^151^ based on read mappings. The regional AQI (R-AQI) scores ranged from 80.89% to 93.33%, and the structural AQI (S-AQI) scores ranged from 56.38% to 71.64% across the assemblies. Assembly completeness was assessed with compleasm (v0.2.7)^152^ using the eudicotyledons_odb12 lineage database. The analysis identified between 95.69% and 99.21% of the expected BUSCO orthologous groups as complete within the assemblies (Table 1). A detailed summary of these genomic features is visualized for the ‘Hanatee’ assembly in Fig. 1.

To evaluate assembly continuity specifically in repetitive regions, we calculated the LTR Assembly Index (LAI). Across the ten assemblies, the adjusted LAI scores ranged from 18.78 to 30.61, indicating assembly qualities spanning high-quality draft (LAI > 10) to reference standard (LAI > 20), with the upper range approaching gold standard quality in terms of intact LTR retrotransposon representation (Table 2).

Finally, we performed Merqury (v1.3) analysis, using a Meryl (v1.3) database constructed from Illumina reads for each assembly, estimated *k*-mer based genome completeness ranging from 71.97% to 89.76% (Table 4). This *k*-mer completeness range is inherently influenced by the nature of these purged, single pseudo-haplotype assemblies. *K*-mers unique to the excluded alternative haplotype are intentionally absent from the reference pseudo-haplotype, thus preventing a 100% representation of all *k*-mers derived from the diploid sequencing reads.

**Table 4:** *K*-mer completeness analysis for pseudo-haplotype *Manihot* assemblies (*k* = 21).

Genome	Heterozygosity (r)	*21*-mer completeness prior to purging (s)	Maximum pseudo-haplotype completeness	Expected pseudo-haplotype completeness	Observed pseudo-haplotype completeness
Hanatee	1.50%	92.9603%	78.65%	73.12%	72.8236%
HighlandRough	1.31%	94.0010%	80.50%	75.67%	73.1022%
HighlandSmooth	1.37%	94.1543%	79.94%	75.27%	71.9676%
Kasetsart50	1.41%	94.5032%	79.52%	75.15%	75.1543%
Rayong9	1.25%	94.0100%	81.16%	76.30%	72.7629%
Rayong72	1.51%	94.0525%	78.52%	73.85%	73.9351%
Rayong90	1.34%	94.9195%	80.22%	76.14%	77.2268%
WhiteRoot	1.39%	94.6117%	79.73%	75.43%	74.1047%
WildType	4.56%	96.3812%	61.65%	59.42%	89.7580%
YellowRoot	1.47%	94.0869%	78.95%	74.28%	73.8572%

The theoretical maximum *k*-mer completeness for an ideal pseudo-haplotype, when measured against the total unique *k*-mers from diploid reads, can be derived from established *k*-mer distribution models in heterozygous genomes^153^. This maximum is given by the formula:

$$Maximum\,pseudo-haplotype\,completeness=1/(2-{(1-r)}^{k})$$

where *r* is the organism’s heterozygosity rate and *k* is the *k*-mer size used in the analysis. For instance, using a *k*-mer size of 21 and representative organismal heterozygosity rates in the range of 0.5%-2.0%, this theoretical maximum *k*-mer completeness would typically fall between 74% (for *r = *2.0%) and 91% (for *r = *0.5%).

It is important to consider that the initial assemblies, prior to purging to create the pseudo-haplotypes, may not have captured the entirety of *k*-mers present in the Illumina reads. This can be due to factors such as incomplete genome coverage by the assembly, sequencing or assembly errors, or challenges in accurately resolving and representing complex heterozygous regions in the diploid state. To account for this, we use the observed *k*-mer completeness of the assembly prior to purging (denoted as scaling factor *s*) as the effective starting fraction of captured diploid *k*-mers. The expected pseudo-haplotype completeness, scaled by this initial capture rate, is then calculated as:

$${Expected\; pseudo}-{haplotype\; completeness}=s\ast {maximum\; pseudo}-{haplotype\; completeness}$$

The observed *k*-mer completeness values for the pseudo-haplotype assemblies generally align well with the expected completeness calculated from the respective pre-purged assembly completeness and heterozygosity. This correspondence suggests that the haplotype purging process was largely effective across these genomes, yielding results consistent with theoretical expectations for single haplotype representation, albeit with minor variations likely reflecting small inefficiencies or specific choices made during the purging process itself. Notably, the *Manihot glaziovii* sample exhibits a significant deviation from this trend. Its observed pseudo-haplotype *k*-mer completeness (89.76%) is substantially higher than both the theoretical maximum for a pseudo-haplotype given its heterozygosity (61.65%) and the scaled expectation based on its pre-purged assembly’s completeness (59.42%). This marked discrepancy strongly suggests that the haplotype purging process was incomplete for this particular, highly heterozygous (4.56%) genome. The retention of a significant portion of both haplotypes is further evidenced by the final ‘purged’ assembly size of 1299 Mbp, which is nearly twice the estimated haploid genome size of 659 Mbp. From this large assembly, only 727 Mbp could be assigned to the 18 chromosomes, indicating substantial unplaced or redundant sequence. Such an inflated assembly size relative to the haploid estimate, coupled with the exceptionally high *k*-mer completeness, indicates that the assembly for *Manihot glaziovii* more closely represents a partially diploid or largely unpurged state rather than a true pseudo-haplotype. The exceptionally high heterozygosity level in *M. glaziovii* WildType is a plausible factor that likely complicated the accurate differentiation and removal of the second haplotype during the purging stage.

The practical implications for users of this specific assembly are significant. The partially diploid nature leads to several unavoidable artifacts: an inflated total genome size (1299 Mbp vs. an estimated 659 Mbp) and gene count (51,770); a high proportion of duplicated gene models, as evidenced by the 70.38% duplicated HOGs in the OMArk analysis (Table 3); and a large amount of sequence (~572 Mbp) that could not be confidently placed onto chromosomes. Researchers should be aware that this redundancy can create challenges for read mapping, variant calling, and comparative genomic analyses, and the data for this specific genome should be interpreted with these limitations in mind.

The corresponding estimated Phred-scaled quality values from the Merqury analysis (QV) ranged from 33.47 to 37.67 across the ten genomes. These QV scores translate directly to high base-level accuracy, indicating estimated consensus error rates between approximately 1 error in 2,220 bases (QV = 33.47) and 1 error in 5,890 bases (QV = 37.67).

Completeness of the gene annotation for each assembly was assessed using OMArk (v0.3.0, OMAmer v2.0.2), PSAURON (v1.0.6), and Mercator4 (v7). OMArk analysis demonstrated that the annotations captured a high proportion of Hierarchical Orthologous Groups (HOGs), with missing HOGs ranging from only 1.69% to 4.18%. However, a substantial proportion of these captured HOGs were identified as duplicates, with duplication rates ranging from 39.85% to 70.38%, while single-copy HOGs ranged from 27.93% to 55.54% across the annotations (Table 3). Complementary analysis with PSAURON indicated high annotation completeness, yielding scores between 97.0 and 97.3 (Table 3). Protein classification via Mercator4 showed that 95.87% to 96.52% of proteins were annotated, with 62.18% to 63.57% being successfully classified into functional bins. Across the assemblies, the annotations covered 93.79% to 96.43% of the Mercator4 BINs (Table 3).

### Limitations of *Ab Initio* Gene Annotation

It is important for users of this dataset to note a key difference in the gene prediction methodologies used. For five of the ten genotypes — Hanatee, Kasetsart50, Rayong9, Rayong72, and WildType — gene prediction was supported by organism-specific RNA-Seq data, which improves the accuracy of gene models. The remaining five genotypes — HighlandSmooth, HighlandRough, Rayong90, WhiteRoot, and YellowRoot — were annotated using only the deep-learning-based *ab initio* tool Helixer. While modern gene predictors like Helixer are powerful, annotations generated without direct transcriptomic evidence are more likely to contain errors, such as incorrect exon boundaries, missed exons, or falsely merged or split genes. We therefore advise that researchers exercise particular caution when analyzing genes from these five genomes, especially in studies focused on rapidly evolving gene families or novel genes, where *ab initio* models may be less reliable.

## Data Availability

The raw genomic DNA (Illumina and ONT) and RNA-Seq sequencing data generated for this study have been deposited in the EMBL-EBI European Nucleotide Archive (ENA) under BioProject accession number PRJEB89494. The final genome assemblies for the ten genotypes are available in the ENA under the individual accession numbers GCA_965363265, GCA_965363285, GCA_965363275, GCA_965363475, GCA_965365905, GCA_965364345, GCA_965364185, GCA_965364695, GCA_965364235, and GCA_965364665. An interactive browser for the assembled genomes and their annotations is accessible at https://www.plabipd.de/ceplas/?config=cassavastore.json. All code used in this project and the final data are available as an ARC repository at PLANTdataHUB^154^ via https://git.nfdi4plants.org/usadellab/Cassava_genome_sequencing_2017.

## Code availability

All code and final data is also available as ARC deposited at PLANTdataHUB^154^ https://git.nfdi4plants.org/usadellab/Cassava_genome_sequencing_2017.

## References

[1] Declaration, F. R. World food summit plan of action. *FAO Rome Italy* (1996).

[2] Fao, F. and agriculture organization of the U. N. FAOSTAT Statistical Database. *Rome URL Httpfaostat Fao OrgendataQCL* **403** (2023).

[3] EL-Sharkawy, M. A. Cassava biology and physiology. *Plant Mol. Biol.* **53**, 621–641 (2003). 15669146 10.1007/s11103-005-2270-7

[4] Nweke, F. I., Spencer, D. S. C. & Lynam, J. K. *The Cassava Transformation*. (Michigan State University Press, 2002).

[5] Olsen, K. M. & Schaal, B. A. Evidence on the origin of cassava: Phylogeography of Manihot esculenta. *Proc. Natl. Acad. Sci.* **96**, 5586–5591 (1999). 10318928 10.1073/pnas.96.10.5586 PMC21904

[6] Olsen, K. M. & Schaal, B. A. Microsatellite variation in cassava (Manihot esculenta, Euphorbiaceae) and its wild relatives: further evidence for a southern Amazonian origin of domestication. *Am. J. Bot.* **88**, 131–142 (2001). 11159133

[7] Prochnik, S. *et al*. The Cassava Genome: Current Progress, Future Directions. *Trop. Plant Biol.* **5**, 88–94 (2012). 22523606 10.1007/s12042-011-9088-z PMC3322327

[8] Bredeson, J. V. *et al*. Sequencing wild and cultivated cassava and related species reveals extensive interspecific hybridization and genetic diversity. *Nat. Biotechnol.* **34**, 562–570 (2016). 27088722 10.1038/nbt.3535

[9] Lyons, J. B. *et al*. Current status and impending progress for cassava structural genomics. *Plant Mol. Biol.* **109**, 177–191 (2022). 33604743 10.1007/s11103-020-01104-w PMC9162999

[10] Landi, M. *et al*. Haplotype-resolved genome of heterozygous African cassava cultivar TMEB117 (Manihot esculenta). *Sci. Data* **10**, 887 (2023). 38071206 10.1038/s41597-023-02800-0 PMC10710486

[11] Awoleye, F., van Duren, M., Dolezel, J. & Novak, F. J. Nuclear DNA content and *in vitro* induced somatic polyploidization cassava (Manihot esculenta Crantz) breeding. *Euphytica* **76**, 195–202 (1994).

[12] Halsey, M. E., Olsen, K. M., Taylor, N. J. & Chavarriaga-Aguirre, P. Reproductive Biology of Cassava (Manihot esculenta Crantz) and Isolation of Experimental Field Trials. *Crop Sci.* **48**, 49–58 (2008).

[13] Wang, W. *et al*. Cassava genome from a wild ancestor to cultivated varieties. *Nat. Commun.* **5**, 5110 (2014). 25300236 10.1038/ncomms6110 PMC4214410

[14] *NCBI Sequence Read Archive.* http://identifiers.org/ncbi/insdc.sra:ERS24509704 (2025).

[15] *NCBI Sequence Read Archive.* http://identifiers.org/ncbi/insdc.sra:ERS24509705 (2025).

[16] *NCBI Sequence Read Archive.* http://identifiers.org/ncbi/insdc.sra:ERS24509706 (2025).

[17] *NCBI Sequence Read Archive.* http://identifiers.org/ncbi/insdc.sra:ERS24509707 (2025).

[18] *NCBI Sequence Read Archive.* http://identifiers.org/ncbi/insdc.sra:ERS24509708 (2025).

[19] *NCBI Sequence Read Archive.* http://identifiers.org/ncbi/insdc.sra:ERS24509709 (2025).

[20] *NCBI Sequence Read Archive.* http://identifiers.org/ncbi/insdc.sra:ERS24509710 (2025).

[21] *NCBI Sequence Read Archive.* http://identifiers.org/ncbi/insdc.sra:ERS24509711 (2025).

[22] *NCBI Sequence Read Archive.* http://identifiers.org/ncbi/insdc.sra:ERS24509712 (2025).

[23] *NCBI Sequence Read Archive.* http://identifiers.org/ncbi/insdc.sra:ERS24509713 (2025).

[24] *NCBI Sequence Read Archive.* http://identifiers.org/ncbi/insdc.sra:ERS24509714 (2025).

[25] *NCBI Sequence Read Archive.* http://identifiers.org/ncbi/insdc.sra:ERS24509715 (2025).

[26] *NCBI Sequence Read Archive.* http://identifiers.org/ncbi/insdc.sra:ERS24509716 (2025).

[27] *NCBI Sequence Read Archive.* http://identifiers.org/ncbi/insdc.sra:ERS24509717 (2025).

[28] *NCBI Sequence Read Archive.* http://identifiers.org/ncbi/insdc.sra:ERS24509718 (2025).

[29] *NCBI Sequence Read Archive.* http://identifiers.org/ncbi/insdc.sra:ERS24509719 (2025).

[30] *NCBI Sequence Read Archive.* http://identifiers.org/ncbi/insdc.sra:ERS24509720 (2025).

[31] *NCBI Sequence Read Archive.* http://identifiers.org/ncbi/insdc.sra:ERS24509721 (2025).

[32] *NCBI Sequence Read Archive.* http://identifiers.org/ncbi/insdc.sra:ERS24509722 (2025).

[33] *NCBI Sequence Read Archive.* http://identifiers.org/ncbi/insdc.sra:ERS24509723 (2025).

[34] *NCBI Sequence Read Archive.* http://identifiers.org/ncbi/insdc.sra:ERS24509724 (2025).

[35] *NCBI Sequence Read Archive.* http://identifiers.org/ncbi/insdc.sra:ERS24509725 (2025).

[36] *NCBI Sequence Read Archive.* http://identifiers.org/ncbi/insdc.sra:ERS24509726 (2025).

[37] *NCBI Sequence Read Archive.* http://identifiers.org/ncbi/insdc.sra:ERS24509727 (2025).

[38] *NCBI Sequence Read Archive.* http://identifiers.org/ncbi/insdc.sra:ERS24509728 (2025).

[39] *NCBI Sequence Read Archive.* http://identifiers.org/ncbi/insdc.sra:ERS24509729 (2025).

[40] *NCBI Sequence Read Archive.* http://identifiers.org/ncbi/insdc.sra:ERS24509730 (2025).

[41] *NCBI Sequence Read Archive.* http://identifiers.org/ncbi/insdc.sra:ERS24509731 (2025).

[42] *NCBI Sequence Read Archive.* http://identifiers.org/ncbi/insdc.sra:ERS24509732 (2025).

[43] *NCBI Sequence Read Archive.* http://identifiers.org/ncbi/insdc.sra:ERS24509733 (2025).

[44] *NCBI Sequence Read Archive.* http://identifiers.org/ncbi/insdc.sra:ERS24509734 (2025).

[45] *NCBI Sequence Read Archive.* http://identifiers.org/ncbi/insdc.sra:ERS24509735 (2025).

[46] *NCBI Sequence Read Archive.* http://identifiers.org/ncbi/insdc.sra:ERS24509736 (2025).

[47] *NCBI Sequence Read Archive.* http://identifiers.org/ncbi/insdc.sra:ERS24509737 (2025).

[48] *NCBI Sequence Read Archive.* http://identifiers.org/ncbi/insdc.sra:ERS24509738 (2025).

[49] *NCBI Sequence Read Archive.* http://identifiers.org/ncbi/insdc.sra:ERS24509739 (2025).

[50] *NCBI Sequence Read Archive.* http://identifiers.org/ncbi/insdc.sra:ERS24509740 (2025).

[51] *NCBI Sequence Read Archive.* http://identifiers.org/ncbi/insdc.sra:ERS24509741 (2025).

[52] *NCBI Sequence Read Archive.* http://identifiers.org/ncbi/insdc.sra:ERS24509742 (2025).

[53] *NCBI Sequence Read Archive.* http://identifiers.org/ncbi/insdc.sra:ERS24509743 (2025).

[54] *NCBI Sequence Read Archive.* http://identifiers.org/ncbi/insdc.sra:ERS24509744 (2025).

[55] *NCBI Sequence Read Archive.* http://identifiers.org/ncbi/insdc.sra:ERS24509745 (2025).

[56] *NCBI Sequence Read Archive.* http://identifiers.org/ncbi/insdc.sra:ERS24509746 (2025).

[57] *NCBI Sequence Read Archive.* http://identifiers.org/ncbi/insdc.sra:ERS24509747 (2025).

[58] *NCBI Sequence Read Archive.* http://identifiers.org/ncbi/insdc.sra:ERS24509748 (2025).

[59] *NCBI Sequence Read Archive.* http://identifiers.org/ncbi/insdc.sra:ERS24509749 (2025).

[60] *NCBI Sequence Read Archive.* http://identifiers.org/ncbi/insdc.sra:ERS24509750 (2025).

[61] *NCBI Sequence Read Archive.* http://identifiers.org/ncbi/insdc.sra:ERS24509751 (2025).

[62] *NCBI Sequence Read Archive.* http://identifiers.org/ncbi/insdc.sra:ERS24509752 (2025).

[63] *NCBI Sequence Read Archive.* http://identifiers.org/ncbi/insdc.sra:ERS24509753 (2025).

[64] *NCBI Sequence Read Archive.* http://identifiers.org/ncbi/insdc.sra:ERS24509754 (2025).

[65] *NCBI Sequence Read Archive.* http://identifiers.org/ncbi/insdc.sra:ERS24509755 (2025).

[66] *NCBI Sequence Read Archive.* http://identifiers.org/ncbi/insdc.sra:ERS24509756 (2025).

[67] *NCBI Sequence Read Archive.* http://identifiers.org/ncbi/insdc.sra:ERS24509757 (2025).

[68] *NCBI Sequence Read Archive.* http://identifiers.org/ncbi/insdc.sra:ERS24509758 (2025).

[69] *NCBI Sequence Read Archive.* http://identifiers.org/ncbi/insdc.sra:ERS24509759 (2025).

[70] *NCBI Sequence Read Archive.* http://identifiers.org/ncbi/insdc.sra:ERS24509760 (2025).

[71] *NCBI Sequence Read Archive.* http://identifiers.org/ncbi/insdc.sra:ERS24509761 (2025).

[72] *NCBI Sequence Read Archive.* http://identifiers.org/ncbi/insdc.sra:ERS24509762 (2025).

[73] *NCBI Sequence Read Archive.* http://identifiers.org/ncbi/insdc.sra:ERS24509763 (2025).

[74] *NCBI Sequence Read Archive.* http://identifiers.org/ncbi/insdc.sra:ERS24509764 (2025).

[75] *NCBI Sequence Read Archive.* http://identifiers.org/ncbi/insdc.sra:ERS24509765 (2025).

[76] *NCBI Sequence Read Archive.* http://identifiers.org/ncbi/insdc.sra:ERS24509766 (2025).

[77] *NCBI Sequence Read Archive.* http://identifiers.org/ncbi/insdc.sra:ERS24509767 (2025).

[78] *NCBI Sequence Read Archive.* http://identifiers.org/ncbi/insdc.sra:ERS24509768 (2025).

[79] *NCBI Sequence Read Archive.* http://identifiers.org/ncbi/insdc.sra:ERS24509769 (2025).

[80] *NCBI Sequence Read Archive.* http://identifiers.org/ncbi/insdc.sra:ERS24509770 (2025).

[81] *NCBI Sequence Read Archive.* http://identifiers.org/ncbi/insdc.sra:ERS24509771 (2025).

[82] *NCBI Sequence Read Archive.* http://identifiers.org/ncbi/insdc.sra:ERS24509772 (2025).

[83] *NCBI Sequence Read Archive.* http://identifiers.org/ncbi/insdc.sra:ERS24509773 (2025).

[84] *NCBI Sequence Read Archive.* http://identifiers.org/ncbi/insdc.sra:ERS24509774 (2025).

[85] *NCBI Sequence Read Archive.* http://identifiers.org/ncbi/insdc.sra:ERS24509775 (2025).

[86] *NCBI Sequence Read Archive.* http://identifiers.org/ncbi/insdc.sra:ERS24509776 (2025).

[87] *NCBI Sequence Read Archive.* http://identifiers.org/ncbi/insdc.sra:ERS24509777 (2025).

[88] *NCBI Sequence Read Archive.* http://identifiers.org/ncbi/insdc.sra:ERS24509778 (2025).

[89] *NCBI Sequence Read Archive.* http://identifiers.org/ncbi/insdc.sra:ERS24509779 (2025).

[90] *NCBI Sequence Read Archive.* http://identifiers.org/ncbi/insdc.sra:ERS24509780 (2025).

[91] *NCBI Sequence Read Archive.* http://identifiers.org/ncbi/insdc.sra:ERS24509781 (2025).

[92] *NCBI Sequence Read Archive.* http://identifiers.org/ncbi/insdc.sra:ERS24509782 (2025).

[93] *NCBI Sequence Read Archive.* http://identifiers.org/ncbi/insdc.sra:ERS24509783 (2025).

[94] *NCBI Sequence Read Archive.* http://identifiers.org/ncbi/insdc.sra:ERS24509784 (2025).

[95] *NCBI Sequence Read Archive.* http://identifiers.org/ncbi/insdc.sra:ERS24509785 (2025).

[96] *NCBI Sequence Read Archive.* http://identifiers.org/ncbi/insdc.sra:ERS24509786 (2025).

[97] *NCBI Sequence Read Archive.* http://identifiers.org/ncbi/insdc.sra:ERS24509787 (2025).

[98] *NCBI Sequence Read Archive.* http://identifiers.org/ncbi/insdc.sra:ERS24509788 (2025).

[99] *NCBI Sequence Read Archive.* http://identifiers.org/ncbi/insdc.sra:ERS24509789 (2025).

[100] *NCBI Sequence Read Archive.* http://identifiers.org/ncbi/insdc.sra:ERS24509790 (2025).

[101] *NCBI Sequence Read Archive.* http://identifiers.org/ncbi/insdc.sra:ERS24509791 (2025).

[102] *NCBI Sequence Read Archive.* http://identifiers.org/ncbi/insdc.sra:ERS24509792 (2025).

[103] *NCBI Sequence Read Archive.* http://identifiers.org/ncbi/insdc.sra:ERS24509793 (2025).

[104] *NCBI Sequence Read Archive.* http://identifiers.org/ncbi/insdc.sra:ERS24509794 (2025).

[105] *NCBI Sequence Read Archive.* http://identifiers.org/ncbi/insdc.sra:ERS24509795 (2025).

[106] *NCBI Sequence Read Archive.* http://identifiers.org/ncbi/insdc.sra:ERS24509796 (2025).

[107] *NCBI Sequence Read Archive.* http://identifiers.org/ncbi/insdc.sra:ERS24509797 (2025).

[108] *NCBI Sequence Read Archive.* http://identifiers.org/ncbi/insdc.sra:ERS24509798 (2025).

[109] *NCBI Sequence Read Archive.* http://identifiers.org/ncbi/insdc.sra:ERS24509799 (2025).

[110] *NCBI Sequence Read Archive.* http://identifiers.org/ncbi/insdc.sra:ERS24509800 (2025).

[111] *NCBI Sequence Read Archive.* http://identifiers.org/ncbi/insdc.sra:ERS24509801 (2025).

[112] Marçais, G. & Kingsford, C. A fast, lock-free approach for efficient parallel counting of occurrences of k-mers. *Bioinformatics* **27**, 764–770 (2011). 21217122 10.1093/bioinformatics/btr011 PMC3051319

[113] Ranallo-Benavidez, T. R., Jaron, K. S. & Schatz, M. C. GenomeScope 2.0 and Smudgeplot for reference-free profiling of polyploid genomes. *Nat. Commun.* **11**, 1432 (2020). 32188846 10.1038/s41467-020-14998-3 PMC7080791

[114] Kolmogorov, M., Yuan, J., Lin, Y. & Pevzner, P. A. Assembly of long, error-prone reads using repeat graphs. *Nat. Biotechnol.* **37**, 540–546 (2019). 30936562 10.1038/s41587-019-0072-8

[115] Hu, J. *et al*. NextDenovo: an efficient error correction and accurate assembly tool for noisy long reads. *Genome Biol.* **25**, 107 (2024). 38671502 10.1186/s13059-024-03252-4 PMC11046930

[116] Rhie, A., Walenz, B. P., Koren, S. & Phillippy, A. M. Merqury: reference-free quality, completeness, and phasing assessment for genome assemblies. *Genome Biol.* **21**, 245 (2020). 32928274 10.1186/s13059-020-02134-9 PMC7488777

[117] Guan, D. *et al*. Identifying and removing haplotypic duplication in primary genome assemblies. *Bioinformatics* **36**, 2896–2898 (2020). 31971576 10.1093/bioinformatics/btaa025 PMC7203741

[118] Chakraborty, M., Baldwin-Brown, J. G., Long, A. D. & Emerson, J. J. Contiguous and accurate *de novo* assembly of metazoan genomes with modest long read coverage. *Nucleic Acids Res.* **44**, e147–e147 (2016). 27458204 10.1093/nar/gkw654 PMC5100563

[119] Alonge, M. *et al*. Automated assembly scaffolding using RagTag elevates a new tomato system for high-throughput genome editing. *Genome Biol.* **23**, 258 (2022). 36522651 10.1186/s13059-022-02823-7 PMC9753292

[120] Hu, J., Fan, J., Sun, Z. & Liu, S. NextPolish: a fast and efficient genome polishing tool for long-read assembly. *Bioinformatics* **36**, 2253–2255 (2020). 31778144 10.1093/bioinformatics/btz891

[121] Camacho, C. *et al*. BLAST + : architecture and applications. *BMC Bioinformatics* **10**, 421 (2009). 20003500 10.1186/1471-2105-10-421 PMC2803857

[122] *NCBI GenBank.* http://identifiers.org/ncbi/insdc.gca:GCA_965363265 (2025).

[123] *NCBI GenBank.* http://identifiers.org/ncbi/insdc.gca:GCA_965363275 (2025).

[124] *NCBI GenBank.* http://identifiers.org/ncbi/insdc.gca:GCA_965363285 (2025).

[125] *NCBI GenBank.* http://identifiers.org/ncbi/insdc.gca:GCA_965363475 (2025).

[126] *NCBI GenBank.* http://identifiers.org/ncbi/insdc.gca:GCA_965364185 (2025).

[127] *NCBI GenBank.* http://identifiers.org/ncbi/insdc.gca:GCA_965364235 (2025).

[128] *NCBI GenBank.* http://identifiers.org/ncbi/insdc.gca:GCA_965364345 (2025).

[129] *NCBI GenBank.* http://identifiers.org/ncbi/insdc.gca:GCA_965364665 (2025).

[130] *NCBI GenBank.* http://identifiers.org/ncbi/insdc.gca:GCA_965364695 (2025).

[131] *NCBI GenBank.* http://identifiers.org/ncbi/insdc.gca:GCA_965365905 (2025).

[132] Ou, S. *et al*. Benchmarking transposable element annotation methods for creation of a streamlined, comprehensive pipeline. *Genome Biol.* **20**, 275 (2019). 31843001 10.1186/s13059-019-1905-y PMC6913007

[133] Beier, S., Thiel, T., Münch, T., Scholz, U. & Mascher, M. MISA-web: a web server for microsatellite prediction. *Bioinformatics* **33**, 2583–2585 (2017). 28398459 10.1093/bioinformatics/btx198 PMC5870701

[134] Ou, S., Chen, J. & Jiang, N. Assessing genome assembly quality using the LTR Assembly Index (LAI). *Nucleic Acids Res.* **46**, e126–e126 (2018). 30107434 10.1093/nar/gky730 PMC6265445

[135] Brown, M. R., Manuel Gonzalez de La Rosa, P. & Blaxter, M. tidk: a toolkit to rapidly identify telomeric repeats from genomic datasets. *Bioinformatics* **41**, btaf049 (2025). 39891350 10.1093/bioinformatics/btaf049 PMC11814493

[136] Kim, D., Paggi, J. M., Park, C., Bennett, C. & Salzberg, S. L. Graph-based genome alignment and genotyping with HISAT2 and HISAT-genotype. *Nat. Biotechnol.* **37**, 907–915 (2019). 31375807 10.1038/s41587-019-0201-4 PMC7605509

[137] Danecek, P. *et al*. Twelve years of SAMtools and BCFtools. *GigaScience* **10**, giab008 (2021). 33590861 10.1093/gigascience/giab008 PMC7931819

[138] Kovaka, S. *et al*. Transcriptome assembly from long-read RNA-seq alignments with StringTie2. *Genome Biol.* **20**, 278 (2019). 31842956 10.1186/s13059-019-1910-1 PMC6912988

[139] Holst, F. *et al*. Helixer–*de novo* Prediction of Primary Eukaryotic Gene Models Combining Deep Learning and a Hidden Markov Model. bioRxiv 2023.02.06.527280 10.1101/2023.02.06.527280 (2023). 10.1038/s41592-025-02939-1 PMC13076211 41286201

[140] Venturini, L., Caim, S., Kaithakottil, G. G., Mapleson, D. L. & Swarbreck, D. Leveraging multiple transcriptome assembly methods for improved gene structure annotation. *GigaScience* **7**, giy093 (2018). 30052957 10.1093/gigascience/giy093 PMC6105091

[141] Schwacke, R. *et al*. MapMan4: A Refined Protein Classification and Annotation Framework Applicable to Multi-Omics Data Analysis. *Plant Syst. Biol.* **12**, 879–892 (2019). 10.1016/j.molp.2019.01.003 30639314

[142] The UniProt Consortium. UniProt: the Universal Protein Knowledgebase in 2025. *Nucleic Acids Res.* **53**, D609–D617 (2025). 39552041 10.1093/nar/gkae1010 PMC11701636

[143] Manni, M., Berkeley, M. R., Seppey, M., Simão, F. A. & Zdobnov, E. M. BUSCO Update: Novel and Streamlined Workflows along with Broader and Deeper Phylogenetic Coverage for Scoring of Eukaryotic, Prokaryotic, and Viral Genomes. *Mol. Biol. Evol.* **38**, 4647–4654 (2021). 34320186 10.1093/molbev/msab199 PMC8476166

[144] Nevers, Y. *et al*. Quality assessment of gene repertoire annotations with OMArk. *Nat. Biotechnol.* **43**, 124–133 (2025). 38383603 10.1038/s41587-024-02147-w PMC11738984

[145] Rossier, V., Warwick Vesztrocy, A., Robinson-Rechavi, M. & Dessimoz, C. OMAmer: tree-driven and alignment-free protein assignment to subfamilies outperforms closest sequence approaches. *Bioinformatics* **37**, 2866–2873 (2021). 33787851 10.1093/bioinformatics/btab219 PMC8479680

[146] Sommer, M. J., Zimin, A. V. & Salzberg, S. L. PSAURON: a tool for assessing protein annotation across a broad range of species. *NAR Genomics Bioinforma.* **7**, lqae189 (2025). 10.1093/nargab/lqae189 PMC11704789 39781514

[147] *NCBI Sequence Read Archive* https://identifiers.org/ncbi/insdc.sra:ERP172520 (2025).

[148] Diesh, C. *et al*. JBrowse 2: a modular genome browser with views of synteny and structural variation. *Genome Biol.* **24**, 74 (2023). 37069644 10.1186/s13059-023-02914-z PMC10108523

[149] Vasimuddin, M., Misra, S., Li, H. & Aluru, S. Efficient Architecture-Aware Acceleration of BWA-MEM for Multicore Systems. in *2019 IEEE International Parallel and Distributed Processing Symposium (IPDPS)* 314–324. 10.1109/IPDPS.2019.00041 (2019).

[150] Li, H. New strategies to improve minimap2 alignment accuracy. *Bioinformatics* **37**, 4572–4574 (2021). 34623391 10.1093/bioinformatics/btab705 PMC8652018

[151] Li, K., Xu, P., Wang, J., Yi, X. & Jiao, Y. Identification of errors in draft genome assemblies at single-nucleotide resolution for quality assessment and improvement. *Nat. Commun.* **14**, 6556 (2023). 37848433 10.1038/s41467-023-42336-w PMC10582259

[152] Huang, N. & Li, H. compleasm: a faster and more accurate reimplementation of BUSCO. *Bioinformatics* **39**, btad595 (2023). 37758247 10.1093/bioinformatics/btad595 PMC10558035

[153] Vurture, G. W. *et al*. GenomeScope: fast reference-free genome profiling from short reads. *Bioinformatics* **33**, 2202–2204 (2017). 28369201 10.1093/bioinformatics/btx153 PMC5870704

[154] Weil, H. L. *et al*. PLANTdataHUB: a collaborative platform for continuous FAIR data sharing in plant research. *Plant J.* **116**, 974–988 (2023). 37818860 10.1111/tpj.16474

